# Rethinking Palliative Care Through Three Institutional Ethnographic Stories of People Living With Homelessness and Life-Limiting Illness

**DOI:** 10.1177/00469580251390760

**Published:** 2025-11-01

**Authors:** Courtney R. Petruik, Katrina Milaney

**Affiliations:** 1 University of Calgary, AB, Canada

**Keywords:** palliative care, end-of-life care, homelessness, institutional ethnography, qualitative research

## Abstract

Fifteen to thirty percent of Canadians have access to palliative care, with even fewer access opportunities for people with experiences of homelessness. Existing research identifies barriers to access but rarely shows how health and social institutions actively organize exclusion. Part of a larger study, this paper examines how health and social systems shape the need for community-based palliative and end-of-life care, using 3 stories from clients of the Community Allied Mobile Palliative Partnership (CAMPP). Using institutional ethnography, data were collected between Fall 2019 and Summer 2020. Sources included approximately 100 h of observation of the CAMPP team’s work, 3 in-depth client interviews, and supplementary provider interviews. Data were analyzed to trace institutional processes that shape everyday experiences of illness and care. Findings reveal systemic demands like renewing insurance for medical equipment, restrictive housing rules, and standardized hospital protocols that overwhelm capacities of many people with experiences of homelessness. Rowan’s story illustrates how bureaucratic requirements jeopardized his oxygen supply. Harriet’s story shows the harm of being separated from her caregiver in housing and hospital contexts compounding distress and reluctance to receive care. Chapa’s story demonstrates how fear and stigma delayed critical cardiology care. Overall, the clients valued CAMPP’s persistent, relational, non-judgmental, and flexible approach. The team’s independence from the mainstream health system mandates enabled responsive care but relies on precarious funding, constraining sustainability. Community-based palliative teams like CAMPP fill critical gaps in mainstream services by tailoring care to complex social realities. Their model shows the value of equity-informed, relational approaches, yet structural exclusion and precarious funding threaten long-term viability. Policy integration must sustain such programs without eroding the autonomy that enables them to deliver meaningful palliative and end-of-life care for people with experiences of homelessness.

## Introduction

In Canada, we have a publicly administered healthcare system and equitable access to it is a public health issue. Fifteen to thirty percent of Canadians have access to palliative care.^1,2^ The World Health Organization^1^ defines palliative care as “an approach that improves the quality of life of patients and their families facing the problems associated with life-threatening illness. [It] prevents and relieves suffering through the early identification, correct assessment, and treatment of pain and other problems, whether physical, psychological, and spiritual.” “End-of-life care” refers to “care administered to patients and their families when patients are approaching death.”^2^

Alberta, Canada has a unique social, economic, and political climate and is facing housing and healthcare crises. Some researchers view these issues as inextricable, given that homelessness is a public health concern.^3^ This paper makes explicit the complexities of managing life-limiting illness in Alberta, Canada, among people with experiences of homelessness through 3 institutional ethnographic stories highlighting the need for a specialized approach to palliative and end-of-life care.

Scholars suggest that early palliative and end-of-life care can improve symptom management, enhance patient and family satisfaction, reduce caregiver burden, and lead to healthcare cost savings.^4,5^ Ideally, palliative care should start close to when a physician diagnoses a patient with a life-limiting illness, but most people who meet the criteria for palliative care receive it in the final days or weeks of life, classifying it as end-of-life care.^6^ For people experiencing housing instability with life-limiting illnesses, access problems are amplified.^6-8^ People with experiences of homelessness report discrimination from healthcare providers, deterring them from services that are available to them.^9^ Studies show that, despite the high prevalence of terminal illness and early mortality rates of the population, people with experiences of homelessness often go without palliative and end-of-life care.^8,10-13^ Homelessness creates a vulnerability that negatively affects health, and when people with experiences of homelessness are also in need of palliative care, they become “doubly vulnerable” (p. 293).^12^

Whether the system repels people or tenuously hooks them in because of precarious situations, publicly delivered services are inadequate for people with experiences of homelessness at end-of-life. In recent years, scholars, practitioners, and policymakers have gained traction in a movement toward equity in palliative care, aiming to help people who are socially marginalized access palliative care when they need it. Scholars argue that the current “one-size-fits-all” approach to palliative care assumes it serves a “standard patient,” a trope rooted in white, middle-class, cultural, and religious values, excluding anyone who does not fit those norms.^14^

An “equity turn” (pp. 2-3)^15^ in health research has recognized the problematic relationship between people with experiences of homelessness and healthcare services. Equitable care aims to “remove unjust and unnecessary differences, requiring us to consider the possibility of making different arrangements for resource allocation, or social institutions or policies.” “Equity-informed” palliative care “sheds light on an array of experiences allowing for a nuanced understanding regarding who current palliative care programs are working for, and who they are failing” (p. 200).^12^ Recently, palliative care professionals have developed community-based teams that work at varying degrees of connectedness to mainstream health systems because they recognized the system was not meeting this population’s needs. This study focuses on one philanthropically funded team located in Alberta, Canada, operating outside the mainstream healthcare system.

Many studies, as typical in nursing, medical, and social services research (a selection cited below), focus on barriers to accessing palliative and end-of-life care and provide recommendations for improved care,^8,13,16-19^ models of care and roles in care delivery,^18,20,21^ preferences and desires of people with experiences of homelessness nearing end-of-life,^22,23^ and advance care planning.^22-24^ However, to our knowledge, no studies examine these topics at the institutional level, showing through client stories, how multiple structures and systems interface to *create* challenges for people with experiences of homelessness requiring palliative and end-of-life care. The current research addresses this gap.

This paper reports on findings from a larger doctoral dissertation study that looked at (1) what clients do to manage their conditions, (2) how they navigate their healthcare needs, (3) what clients value about community-based palliative and end-of-life care, and (4) what enables or constrains the program to work in their specific way. While experiences of people who have been or are homeless are at the center of our study, clients are not meant to be the analytic object. Through their stories, we focus on what warrants the practice of a different type of palliative care.

### The Community Allied Mobile Palliative Partnership (CAMPP)

In Alberta, Canada, a community-based non-profit palliative and end-of-life care team exists called the CAMPP. At the time of data collection, the core team comprised 3 people operating from a community health clinic: a nurse coordinator, a health navigator, and a palliative care physician. All clients were people with experiences of homelessness and a life-limiting diagnosis. At the time of research, philanthropic organizations (ie, grants, donations, in-kind support) funded the CAMPP team rather than receiving funding from the provincial health ministry.

A CAMPP team program evaluation^20^ found that they served 128 clients, with 29 identifying as female and 99 as male with the average client age as 58.9 years (ranging from 22 to 86). The most common primary diagnosis for CAMPP clients was cancer. Most referrals to the CAMPP team came from community partners, acute care, and their home agency. Common barriers for people referred to the CAMPP team included absence of a primary care physician, unstable housing, low/no income, insufficient medication coverage, and lack of social supports. The evaluation showed that the CAMPP team supported clients with housing as a care priority, which was notable given that they are healthcare providers, an atypical priority in current systems tending toward a biomedical model. The CAMPP team also supported clients in *sustaining* housing and navigating problems. CAMPP clients entered the program homeless, but over time occupied all points on the housing spectrum, including living on the streets, in emergency shelters, in transitional housing, couch-surfing, long-term stays in hospitals, or in their own private dwellings.

## Methods

### Theoretical Framework

This study used institutional ethnography (IE) as its mode of inquiry.^25^ IE does not theorize about a social world that exists “out there” but is rooted in the ontological position that we find our knowledge about social occurrences in what people are *doing, thinking, seeing, and imagining.* IE explicates what is not directly visible to us in our everyday lives by tracking interactions, actions, activities, and tasks of people and linking up relevant texts through people’s practices to “map” how the social world is organized. IE is a “mode of inquiry” because it encompasses *both* a methodology and a theoretical approach.^26^ IE differs from traditional ethnographies as it pursues analysis on multiple levels of social organization through collection at multiple sites and “beyond what informants at the local level may know” (p. 81).^27^ IE confronts relations occurring between people that shape or are shaped by social inequities.

### Research Objective

Through the access point of CAMPP clients, we show how institutions “create the conditions of individual experience.”^28^ Our approach contrasts other methodologies, such as narrative research, which focuses on personal meaning-making. The aim of this paper is to show, through 3 client stories, what necessitates the need for specialized community palliative care for people with experiences of homelessness. This study gained ethical approval from the University of Calgary Conjoint Faculties Research Ethics Board in 2019 (Ethics ID: REB19-0337). Further, this study followed the Standards for Reporting Qualitative Research guidelines.^29^

### Data Collection

#### Observations and Interviews

Commonly, IE starts with observations of practices and activities^25^ moving iteratively throughout data collection. Between Fall 2019 and Summer 2020, we accumulated approximately 100 h of observations on the road, in meetings, and in care settings. The observations focused on the organization of the CAMPP team, how they conducted their work, and how the processes and structures of multiple intersecting systems (eg, healthcare, social services, private vendors) shape clients’ experiences. We chose “threads of interest” from the observations, areas in which to do a “deeper dive” via interviews based on what came up often and was important to participants.^26^ The data guided the questions we asked interviewees.

While IE does not typically follow a structured interview guide, we included open-ended topics in the interviews that were grounded in participants’ experiences. The initial questions focused on everyday activities and challenges, with follow-up questions shaped by participants’ responses and emerging institutional processes. We conducted 3 in-depth semi-structured interviews with the intent to capture rich and in-depth client accounts, not to generalize to every person who experiences homelessness and a life-limiting illness. As part of the larger study, we also conducted interviews with CAMPP staff and adjacent service providers and administrators. With CAMPP team caseloads of approximately 25 clients, the total population of potential interviewees is small, with fewer people who were in stable enough condition for an interview. We recruited client interviewees via the CAMPP team, who informed clients of the study, and if they expressed interest, the team would request consent for a researcher to contact them, and the researcher would arrange participation. This process ensured that the clients did not feel pressure to participate and assured that their participation (or not) would not affect their care. A member of the research team reviewed consent forms with interviewees and, depending on the preference of the interviewees, recorded written or verbal consent.

### Analytic Approach

As equity-oriented researchers, our personal and professional experiences inform our approach and assumptions. Our academic training is in sociology and public health, and we bring personal insight and scholarly perspective to this work. We engaged in reflexive practices throughout the study, writing analytic memos, holding debriefing sessions with the research team, and having discussions that critically examined how our social locations, values, and relationships with participants have shaped the research process and interpretations of the findings.

The interviews were audio recorded and transcribed verbatim with identifying information removed. The first author analyzed and wrote up the data as a compilation of the observations and interviews. The second author reviewed and provided feedback on the analysis and write-up of the study. We used NVIVO 12 Qualitative software to support analysis.

Three client interviews formed the base of the findings; however, observations informed the direction of the interviews and formed sections of the story content, as the clients were interviewees but also present during observations. We “indexed” the data referring to a way of thinking that helps avoid analytically *drifting* from focusing on the “institution to the informants” (p. 4).^30^ Indexing, typical in IE, is a tool to cross-reference between people and their practices as they interface in their activities. It is important that the researcher takes the meaning of the indexed content in relation to its context. This involved reviewing all the data (ie, observations, memos, transcripts, and program documents) to find instances where clients spoke about their experiences managing their health or navigating health care. We then took all related practices across data sources and placed them together under headings, including naming how they interface, align, or do not align, with each other. This was done to find points of coordination or broken coordination. If we were uncertain about a linkage or a heading, we made a note and followed up about it in a subsequent observation or follow-up interview. To build the narratives, we started with concrete moments or problems that people experienced as shown in interviews and observations with clients. We then took clauses and excerpts that illustrated how the problems are institutionally organized (ie, pulling in quotes or notes about system demands, expectations, or required texts). All narratives are based in the data and real client experiences.

The analytic object of this investigation is the *practice* of community palliative and end-of-life care to show how different practices are necessary based on how people, socially marginalized by homelessness and life-limiting illness, experience their subjugation. We present our findings as 3 clients’ stories; however, the details of each story highlight the institutional processes that constrain, detach, and deter individuals from receiving care they need based on their social positioning.

Aside from the name of the CAMPP team, because they gave us consent to use it, we removed all identifying information to protect participant anonymity and confidentiality. We interviewed 3 CAMPP clients and combined interview data with relevant observational data to illustrate, grounded in client experience, why and how a team like the CAMPP team is necessary amidst having “publicly delivered” palliative healthcare.

All names in this study are pseudonyms to protect the identity of participants. As is typical in IE, we triangulated data from interviews and from hours of observations to map how institutional structures shape everyday experiences by linking individual accounts to broader systemic forces.^25^ The combination of interviews and observational data into 3 stories allows for a multi-perspective, context-rich understanding of the structural barriers and everyday challenges faced by people with experiences of homelessness served by the CAMPP team. Due to the rapidly changing climate of non-profit organizations in Alberta, Canada, it is possible that some of this data may not reflect the current practices of the CAMPP team.

## Results

### Rowan’s Story: “I Can’t Afford to Breathe”

Rowan is a 70-year-old man who has a severe chronic respiratory illness and uses 2 oxygen machines: a large one and a portable one.

Interviewer: How did you come to know about CAMPP and work with the team?

Rowan (Interviewee): Before I lived here, I was in the hospital and staying at the local shelter. Before that, I was in Saskatchewan for about 10 years. A worker helped me get housing from the hospital; she introduced me to [the CAMPP team].

We spoke about how Rowan and the CAMPP team first connected, and he quickly moved into talking about recent struggles.

Rowan: I was taken off oxygen *three* to *four* months ago, and that put me in the hospital for 10 days. I called [the CAMPP team] yesterday because I got a weird phone call from the oxygen company, so [the CAMPP team] are phoning them for me. I have COPD, which is cardio-obstructive pulmonary disease, a combination of emphysema and bronchitis. I have had it for quite a while, and I have been on oxygen for about *three* years. Ever since I came here, well no, it was when I was in the hospital, and I got home here I needed oxygen, and they came and took it all out so I ended up back in the hospital because they called an ambulance, and I couldn’t breathe. They never told me they were gonna take my oxygen, I came home and no oxygen. They said it wasn’t paid for; it was freaky.

Interviewer: What was the process of getting it back?

Rowan: I had to go to the hospital again. You know what an ambulance is worth? $385.00 *one* way. I have [insurance], but it sure costs the system. There are nurses and orderlies and doctors and they gotta be paid, they are not working on welfare. What does the nurses make? Probably 50 bucks or better 100 bucks an hour.

Retaining oxygen for Rowan involved periodic tests and authorization from his physician, and if those did not happen in time for Rowan to submit them to the government to ensure payment to the oxygen supplier, the vendor repossessed the device. Going to the emergency department was the quickest way for Rowan to get the test and authorization for his oxygen, but as he explained, the cost to the healthcare system is high, complex, and involves many intersecting systems.

Rowan: It doesn’t cost me, but I pay for my meds. I am not on AISH [Assured Income for the Severely Handicapped] because I am 70 but [health insurance] pays some and it pays a bit but I gotta put money out each month. I just paid a big bill at the pharmacy. The last bill was $179.00 and because I don’t have much money right now ‘til I get paid, I gotta wait until I get my old age pension around the end of the month. A couple weeks yet.

Rowan was on a provincial supplemental health insurance plan for people over age 65. Because of the way Canada’s public health insurance system is set-up in Alberta, this meant that he paid up to 30 percent of each prescription to a maximum of $25.00 per medication, and public insurance covered the rest. He paid the difference at the pharmacy but found it difficult given that he is on expensive medications and has a limited income. A challenge was that the insurance coverage for the oxygen expired intermittently and required him to continually update the insurer with oxygen levels to secure eligibility. Normally, Rowan’s respirologist appointments would manage this since they take his oxygen levels and provide them to the health insurance program, which depending on whether he met the requirements, approved the cost of the oxygen machines. He also spoke about upcoming responsibilities, drawing on his knowledge of past challenges and the help available to him.

Rowan: Do you think I should phone [the oxygen vendor] again? I need [the oxygen] if I am going to the store. I can’t walk there, but I hit the foodbank and its next door, they are really helpful, they will bring it right over for me because I am pushing oxygen. They help me quite a bit, so I don’t have to pick up the heavy canned foods. They carry that for me. If I need to get to and from medical appointments, then [CAMPP] helps me and will meet me here and go with me in the cab. It is hard to keep track of all the appointments. If I don’t write it down, I will forget it.

Rowan explained to the interviewer that he was having trouble with appointments. This became apparent during our meeting when he asked for help looking for a phone number that he wrote on a torn off corner of a cigarette carton. The room had items laid about on the tables and floors, so it was challenging to locate a corner of cardboard with a scribbled-down phone number on it. The difficulties he faced accumulated. He had competing items to track despite his limited physical and financial capacity. Shortly into our visit, an oxygen company representative knocked on the door, and Rowan reluctantly answered.

Rowan: [to vendor] *I* need both machines. *I* went outside for 10 minutes, and *I* had to come in just to breathe. *So*, *I* need the big one, but *I* need the small *one* if *I leave* the house. I wouldn’t last to make it to the store even by taxi without it. . .please, you can’t just take them. . .

Vendor Rep: Phone [our office] then.

As Rowan was making the call, I asked the representative what was happening. He explained that the oxygen vendor was seizing Rowan’s machines because his funding was inactive due to missing his last respirologist appointment. Rowan explained that he missed the appointment because he lost track of it. Rowan tried negotiating with the vendor to delay the payment for 2 weeks, but they did not accept this reasoning. Their policy was to secure the payment or remove the tanks, not address personal context.

Rowan’s living conditions made it difficult and sometimes impossible to meet the expectations of interfacing organizations involved with his needs. Despite Rowan’s best efforts, his needs remained unmet.

Rowan: I asked [CAMPP] to come to my respirologist appointment in *two* weeks in a cab. I have $58.00 to my name for *two* weeks until I get paid. . .if they take my oxygen, I can’t afford to breathe.

The CAMPP team planned to attend his next appointment with Rowan. Rowan’s need was imminent; he needed help talking to the vendor because his voice was “running out” due to his illness. Using his available resources, he requested that I call the CAMPP team on his behalf to request that they help him manage the situation. I called the CAMPP team, and after they spoke with the vendor, informed me that the vendor would leave the smaller tank but would confiscate the larger tank. The result dismayed Rowan, though he was relieved that he would have portable oxygen and that, for the time being, he would not have to return to the hospital, something that terrified him given the new and unfamiliar threat of COVID-19. At the time of research, public messaging about COVID-19 was warning immunocompromised elderly people to avoid being in public, and vaccinations were not yet available.

### Harriet’s Story: “Because We Were Homeless, We Were Treated So Badly”

Harriet suffered from a rare disease that affected her kidneys. She was reluctant to seek medical attention because of negative past experiences.

Harriet: Every hospital I stayed in, they would never let [my husband] stay with me because we were homeless, we were treated so badly. At the [hospital], all the husbands get to stay with their wives, or their wives with their husbands, and mine was the only *one* that was not allowed, literally not allowed to stay there. . .and CAMPP had to come and take [my husband] to the cafeteria and bought him something to eat, some food and the nurse, I can’t remember her name, but the nurse, the head nurse, she literally gave him a garbage bag to sleep outside because it was raining. . .he had to sleep outside. It was horrible the way homeless people are treated.

Similarly, her place of residence also prohibited her and her husband from residing together. Harriet explained that she had just moved up to her suite in a subsidized housing program offered by a community organization for people who have been homeless. However, their regulations prohibited them from living in the same suite, separating them and forcing him to travel to her, several times each day to care for her which he sometimes was unable to do, in which case, she called the CAMPP team. Her husband was her main support system, helping her with important tasks such as administering her medications. She explained that it “gets tricky because so many of the pills look alike.” In an observation, there was 1 interaction where she held out her hand for the medications from her partner as she said, “he takes good care of me.” They smiled, exchanged a kiss, then he left the apartment. This echoed an earlier observation where the CAMPP team members selected certain pills out of clients’ bottles and placed them in containers with the first letter of the day of the week on the outside to help them stay organized. This attention to medication was a common service that the CAMPP team provided.

Further, Harriet was reluctant to use healthcare system resources. She waited until her disease progressed until it resulted in an inevitable reliance on the system and a pattern of frequent hospital admissions.

Harriet: When I was on the street, ya know, it got to every *two* days I was back in the hospital, every *two* days by ambulance. . . All I knew is that I needed somebody to come to me and [the CAMPP team] just did. . . There was a time when [the CAMPP team] came every single day, [First Author Name]. There was not *one* day they did not come to see me. It was in the beginning, [the healthcare professionals] did not believe I would live more than *two* weeks, and it has been almost *one* year. It is miraculous.

For Harriet, the CAMPP team’s visits were a significant factor in her living beyond the 2 weeks that she expected when she was in the hospital. Harriet valued the CAMPP team’s approach. She noted a significant part of what made the CAMPP team a unique service that worked for her:

Harriet: All this stuff, the crap we deal with in the hospital is something we never experience with CAMPP. They don’t give a shit what you do or what you’re doing. They are never going to leave you. And they told me that, and you know what? They never have. They are still here. And I’ve told them to go fuck their hats, like literally, ya know, just go fuck off and they still come back. Of course, I apologized, I don’t mean it.

Harriet expressed that a vital component of the CAMPP team’s work was that they persisted in coming back despite her attempts to push them away. In her experience, this persistence and acceptance of her did not exist in the mainstream healthcare system.

### Chapa’s Story: “Judgemental is a Bad, Bad Word. . .”

Chapa’s first meeting with the CAMPP team was at an emergency shelter that he and his wife stayed at intermittently for years. He was frightened to speak to anybody about his “troubled heart” because he did not want to learn that he was dying. After talking with his wife, Chapa reluctantly decided to “trust” the CAMPP team. Chapa explained that the relationship with the team blossomed, and as he began working with them, part of what helped foster trust between them was that he did not feel judged for being homeless.

Chapa: I have a lot of respect for [the CAMPP member]. For caring and showing me that he cares. Judgmental is a bad bad word. . .Here in Calgary, some family members, and some professionals, they won’t rent to us or talk to us because we are homeless you know. They judge us. You know if you have a heart condition like I do it was really hard for me to live that way. Sleep in parkades against those big cement blocks that stop the car. . .you get comfortable and then you hear the guards yell, “you gotta leave!” Because stereotypes. Why should a person have to change themselves?

These acts toward him contrasted with his view of the CAMPP team. Having trust in the team members helped him navigate his fear of his medical appointment.

Chapa: I can open up [with CAMPP] about anything. I trust them. I

was thinking about the time with [staff member] and [CAMPP] and they were talking to me about coming to my heart appointment. It’s way down South and I didn’t want to go. It was far and I didn’t want to find out more bad news, I didn’t want to find out why I am sick and if I can’t get better. I do 99% of the job, the *one* percent, it’s that *one* percent that helped me all the way through. Maybe, if I didn’t take that *one* percent chance with [CAMPP] to go to that appointment I wouldn’t be here.

Chapa described the distress he felt that created barriers to getting care. Chapa explained the location of the appointment was far for him to travel. Having limited access to reliable transportation, minimal income, and being in poor health, a long commute was challenging. The CAMPP team supported him by talking with him through his fears, providing transportation, and accompanying him to his appointment. He described how the cardiologist provided him with a piece of paper that had the physician’s name, location, and time of the appointment, with little other information on it. He explained that this information did not reassure him about his fears. The paper did not help him understand the clinical process or the people that he would be meeting with. Of utmost concern to him was that he did not know the physician he would be meeting with to discuss such personal matters, and this was important to him. Like Harriet, he had little trust in healthcare providers based on previous experiences.

The institutionally embedded processes of informing patients about critical appointments were standard for all patients. While the expectation is that this information is sufficient for attendance, it was insufficient for Chapa, given his experiences. Chapa satisfied the expectation of the healthcare system to meet the critical appointment because he connected with the CAMPP team, who built their model to acknowledge and address Chapa’s challenges. Chapa attended the appointment and expressed that he would not be alive if he had not trusted the CAMPP team.

## Discussion

The client stories demonstrate how the system pushes individuals to navigate intersecting processes that exist across institutions (eg, healthcare, social services, private companies) and how this translates into underrecognized work, concerns, needs, challenges, and unfulfilled needs and preferences related to their care.

Much of the current literature in palliative and end-of-life care and homelessness focus on barriers associated with *accessing* palliative care,^8,13,16-19^ and while this is incredibly useful, the current paper adds, through client stories, what necessitates the need for specialized community palliative care for people with experiences of homelessness.

This research recognizes agency for people with experiences of homelessness with life-limiting illnesses, bringing into view the relations that evoke the need for specific “health work”^31^ in managing their challenges. In this paper, the 3 client stories show how systems work to construct experiences that complicate things for people who have experiences of homelessness and life-limiting illness and how this creates a need for specialized care.

Rowan’s struggle to maintain oxygen eligibility illustrates how the health system outsources administrative care work to patients least able to complete it. The missed appointment resulted in rescheduling, which triggered a sequence of processes resulting in added challenges for Rowan. Rowan relied heavily on a device that he was now without because of a misstep within a complex system of care not built for his circumstances. Rowan’s encounter illustrated a regular interface with multiple institutions: the healthcare system, a government insurance system, the system of private oxygen vendors, transportation systems, banking systems, commerce, and non-profit agencies. Each system had its own standards, procedures, and processes as its priorities that Rowan was expected to navigate. This bureaucratic burden compounds vulnerability, effectively rationing care by one’s capacity to navigate paperwork rather than by needs of the individual. This story demonstrates how institutional processes make survival contingent on administrative vigilance. Missing a single respirology appointment triggered a series of events that resulted in the loss of oxygen access. This illustrates how bureaucratic systems measure eligibility in ways that disregard the precariousness of daily life with homelessness and life-limiting illness. Stajduhar et al describe this as the paradox of being “too busy surviving” to manage the bureaucratic requirements of care.^13^ Rowan activated the tools he had through advocating for himself via attempted negotiations with the vendor, making notes to track his appointments, by contacting the CAMPP team for help, and even asking for guidance from the research team. Perhaps a system that is structured to consider constraints like Rowan’s might benefit not only people with structural vulnerabilities, but also the broader system.^21^

Further, Harriet highlighted how “care work” extends beyond clinical encounters. Her reliance on her spouse as a caregiver was undermined by housing and hospital policies that prohibit cohabitation. This shows how sometimes institutional rules fracture essential care relationships, forcing reliance on external providers like CAMPP to fill the gap. Authors also show that equity failures in palliative care often arise when institutional rules are applied without regard to relational needs.^12^ Harriet’s story highlights the dis-entitlement she felt with the healthcare system. As an experienced patient, Harriet knows what works for her, as shown in her statement above, “all I knew was that I needed somebody to come to me and [the CAMPP team] just did.” She did not want to push them away, but her stressors put her in a negative mental state. This did not mean she wanted to terminate services, but that her reactions were harsher than intended. She appreciated that the CAMPP team did not let her reactions impede her care.

Finally, Chapa’s story illustrates how the avoidance of a cardiology appointment gets transformed into a health-avoidant behavior rather than mistrust of a system that has historically harmed him. Chapa’s expression of doing “99% of the job” orients us to his agency and how he was the main actor in working to manage his health and daily activities but had a challenging time with the final pieces of work. He recognized that he needed the “one percent” support, which was not available to him until he connected with the CAMPP team. His fear was not irrational, it was produced by repeated experiences of judgment and stigma, which made medical engagement unsafe emotionally for him. His eventual agreement to attend, supported by CAMPP, underscores how trust-building becomes life-sustaining care. This reflects findings from scholars, who show that structurally vulnerable people often delay or are reluctant to seek care often due to prior discriminatory encounters.^16,17^ The bureaucratic tools of the health system, the appointment slips and intake forms, for instance, were assumed sufficient, yet failed to meet Chapa’s needs. This reiterates findings from McNeil et al who show how standardized health system processes can exclude people experiencing homelessness by ignoring lived context of stigma and mistrust.^32^

The current research also demonstrated how navigating one’shealthcare needs was also shaped by one’s social position as a person with experiences of homelessness with life-limiting illness. Rowan stitched his care together across fragmented systems doing invisible “coordination work” that is usually managed by professionals. Giesbrecht et al also highlight how structurally vulnerable patients are required to navigate multiple institutional interfaces that were not designed with them in mind.^8^ Harriet’s exclusion of her spouse from both housing and hospital spaces illustrates how policies reproduce social isolation as an administrative outcome that neglects client needs. Scholars have stated this, arguing that Canada’s palliative system often “fails by design,” entrenching inequities for people already marginalized.^19^

Lastly, the 3 client stories also showed what people with experiences of homelessness value about community-based palliative care. Rowan valued CAMPP’s responsiveness and flexibility by having practices that disrupt the rigidities of mainstream systems. Harriet described CAMPP’s persistence and non-judgmental stance as transformative. This echoes other authors who highlight equity-informed palliative care as grounded in relational endurance and the refusal to withdraw care even when clients resist engagement.^12^ Both Chapa and Harriet highlighted the absence of judgment as foundational for care. Studies confirm that stigma is structurally reproduced in healthcare encounters with people experiencing homelessness.^10,25^ CAMPP’s non-judgmental stance actively counters this structural deterrent. Finally, for Chapa, accompaniment to his medical appointments was not only logistical support but reassurance. Another study described accompaniment as an equity intervention redistributing power by standing with clients in exclusionary spaces like some clinical settings.^11^

Rowan, Harriet, and Chapa shared their experiences while managing their health conditions that were complicated by homelessness. All clients in the study experienced forms of discrimination in their encounters with the health system. This follows the literature purporting that experiences of discrimination by healthcare providers toward people with experiences of homelessness are not uncommon and hinder access to care.^9,19^ Despite the systemic ignorance of their needs, clients navigated the complexities of the system with support from CAMPP. In its current form, the healthcare system is based on a “one size fits all” approach in palliative and end-of-life care, excluding the needs of socially vulnerable people,^33^ warranting a different approach to care.

Relying on the CAMPP team’s support with appointment schedules, transportation, and being available when needed, aided individuals in challenging situations. Of key importance through all 3 stories was the lack of negative judgment undergirding the CAMPP team’s approach. Studies have pointed out that a non-judgmental approach to care is key in delivering palliative and end-of-life care.^19,34^ The individuals highly valued the CAMPP team’s lack of judgmental attitudes toward them, something they found imperative to developing and sustaining connections with people who had negative experiences with care providers.

## Moving Forward

While clients and service providers find the CAMPP approach valuable and effective for people with experiences of homelessness,^20^ programs like the CAMPP often cannot be operationalized annually without sustainable funding. The CAMPP team runs as a model external to the mainstream health system, creating challenges in sustaining and expanding the work. The question becomes, if this is an effective and valued service, then why is it not embedded in our current mainstream system? The wider study of which this paper is a part, explores a paradox that this team encountered was one in which they either stay outside the mainstream system to maintain the integrity of their model or be invited into the mainstream system but risk losing the ability to work in the way that they do which is valued and effective for this client population. Similar models in other areas have found ways to conduct this work ensuring sustainability and integrity of the approach (see PEACH in Toronto, Ontario, Canada, for example). However, in Alberta, the socio-political climate and the health system’s current form do not have socially vulnerable populations as a priority. It is vital that we keep working in the direction toward equitable palliative and end-of-life care as people with experiences of homelessness disproportionately carry the burden of illness, mortality, and suffering.^33^ Future research should explore the work of community-based health teams who do work that is highly complex, difficult, and physically and emotionally taxing, yet often goes underfunded and underappreciated.

## Limitations

Limitations of this study included having limited data from people who were actively dying or those not connected to services. We could have strengthened this study by further investigating the mainstream palliative care system and how this work is socially organized. Another limitation of this research is that it did not report on the demographics of interviewees, which limits the depth of analysis, speaking to important intersectional identities such as gender and race. Scholarship should build this into future work and gather evidence to address intersectional inequities in end-of-life care.

## Conclusion

This research has implications for policy and practice in health and social programming. This study is grounded in the voices of people with experiences of homelessness who are oppressed by and often lost in our large and complex health and social care systems. This study builds on the existing literature, carrying the values and goals of the equity in palliative and end-of-life care movement forward, involving people with experiences of homelessness in research, policy, and practice. This research helps identify how to maintain or reproduce effective practices focused on people with experiences of homelessness with the ongoing goal of achieving equity in palliative and end-of-life care. Future research should continue to work with people with experiences of homelessness with life-limiting illness, ensuring the work stays grounded in the lived experiences of those who are most impacted by systemic inequities.
